# Detecting Regions At Risk for Spreading COVID-19 Using Existing Cellular Wireless Network Functionalities

**DOI:** 10.1109/OJEMB.2020.3002447

**Published:** 2020-06-15

**Authors:** Alaa A. R. Alsaeedy, Edwin K. P. Chong

**Affiliations:** Department of Electrical and Computer EngineeringColorado State University 3447 Fort Collins CO 80523 USA

**Keywords:** COVID-19, infectious diseases, tracking

## Abstract

*Goal:* The purpose of this article is to introduce a new strategy to identify areas with high human density and mobility, which are at risk for spreading COVID-19. Crowded regions with actively moving people (called **at-risk** regions) are susceptible to spreading the disease, especially if they contain asymptomatic infected people together with healthy people. *Methods:* Our scheme identifies **at-risk** regions using existing cellular network functionalities—*handover* and *cell (re)selection—used to maintain seamless coverage for mobile end-user equipment (UE)*. The frequency of *handover* and *cell (re)selection* events is highly reflective of the density of mobile people in the area because virtually everyone carries UEs. *Results:* These measurements, which are accumulated over very many UEs, allow us to identify the **at-risk** regions without compromising the privacy and anonymity of individuals. *Conclusions:* The inferred **at-risk** regions can then be subjected to further monitoring and risk mitigation.

## I. Introduction

The global COVID-19 pandemic is easily spread by people in close proximity, especially in crowds with mobile individuals (e.g., city centers). A widely accepted strategy to mitigate its spread is social distancing, avoiding crowded areas [1]. There is an urgent need for different mitigation strategies to slow the spread of this disease. Spreading by “silent carriers” mostly depends on how they move and gather, the two viral-spreading risk factors motivating our new mitigation strategy.

Our strategy does not track individuals, unlike many existing contact-tracing mobile-phone apps [2], which require widespread user adoption and have obvious privacy concerns. Instead, we anonymously measure the aggregate density and mobility of mobile devices, without individual identities, as detailed below. Moreover, these measurements do not require installation of any app nor any other action on the part of mobile users.

## II. Materials and Methods

We exploit already existing cellular-network functionalities intended to manage end-users’ mobility and to ensure seamless coverage [3]. Because practically everyone carries cellular mobile devices (called *user equipment (UE)*), these serve as always-on human trackers. More specifically, the higher the number and mobility of UEs, the higher the number and mobility of people.

According to a recent study [4], SARS-CoV-2 can live in the air for up to three hours (remaining viable in aerosols), exhaled by infected people while speaking, coughing, or even breathing, whether symptomatic or not [5]. We are particularly concerned with the scenario where contagious people are present in areas with many other continuously mobile people. Such areas, which we call **at-risk**, naturally have high local basic reproduction number ($R_0$) [6]. Conversely, sparse areas with mostly stationary people are not considered **at-risk** (e.g., residential areas with people remaining at home). The main goal is to detect **at-risk** areas, allowing prioritization for further monitoring and risk management. Our strategy is based on inferring the crowdedness and mobility using measurements of quantities already accessible to the cellular wireless network via UE mobility management protocols.

### A. UE Mobility Management

Our scheme detects **at-risk** regions by measuring UE mobility and density over a day or more, to capture long-term behavior rather than short-term transients. Specifically, we exploit existing network functionalities required to keep each UE connected while moving, exchanging UE-specific information with the network [7], as detailed below.

### B. Handover and Cell Selection

*Long Term Evolution* (LTE) networks (and their 5G successors) have shifted toward ultra-dense small-cell deployment, called Heterogeneous Networks (HetNets), comprising multiple layers of different cell sizes: microcell, picocell, and femtocell; see Table I [3]. HetNets need to accommodate the increasing density of highly mobile UEs and keep power consumption low in battery-limited UEs [8]—hence, small cells are deployed in dense UE areas.

**TABLE I:** Cell Types in Cellular Networks (Adapted From [3])

Cell type	Coverage range (meter)
Femtocell	10–20
Picocell	200
Microcell	2000

The mobility of each UE is handled by two essential protocols: *handover (HO)* and *cell (re)selection (CS)* [9]. We use the measurements from *conventional* *HO/CS* events only, intended to handle moving pedestrians as they cross cell boundaries. We exclude the *HO/CS* events triggered by moving vehicles, handled by different procedures called *fast* *HO/CS* [10], who do not contribute significantly to spreading COVID-19. Each UE.^1^^1^While moving, the UE triggers *HO* when it is in the CONNECTED state and *CS* when it is in the IDLE state [11]. triggers these procedures while moving from one cell to another (e.g., from femtocell to picocell), to maintain connectivity. As UE density and mobility increases, so does the rate of *HO/CS* events. Thus, measuring *HO/CS* rates can be used to identify regions with high UE density and mobility, thereby identifying **at-risk** regions. The higher the *HO/CS* rates, the higher the risk of spreading COVID-19. Because crowded areas are likely to have small cell sizes, the spatial resolution of the *HO/CS* measurements is also relatively high (10–20 meters in femtocells). Continuously measuring *HO/CS* rates gives real-time updates of regional **at-risk** status.

## III. Results

**Fig. 1.**
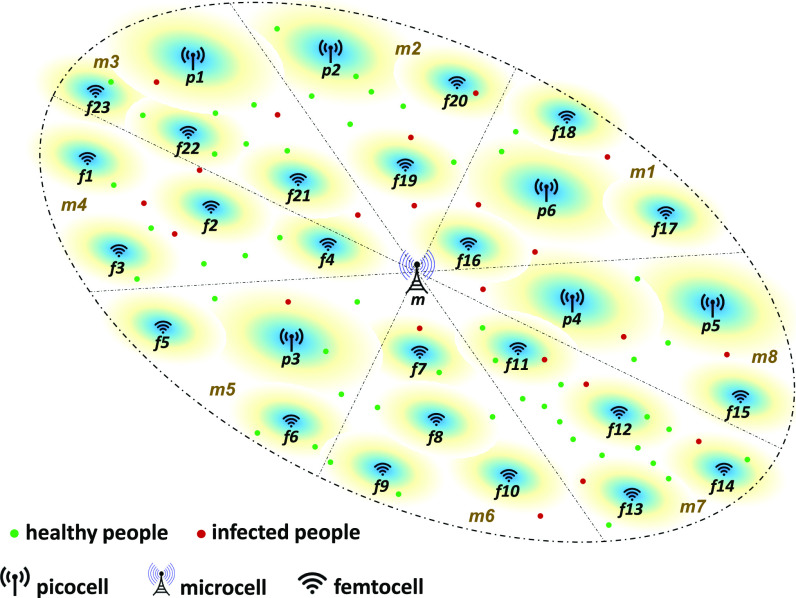
Illustration of HetNet deployment in areas with healthy and infected people.

Fig. 1 depicts an example of multiple cell sizes of a HetNet, deployed according to a predefined network plan; i.e., where these cells are needed to accommodate UE connectivity in high-density areas. While actively moving, UEs frequently initiate *HO/CS* events. Typically, each cell (labeled m, p, and f in Fig. 1) records these events to be used by the network as key performance indicators (KPIs) [7], primary indicators used to evaluate and measure network performance; e.g., *handover success/failure rate*.

If the *HO/CS* rates from a certain cell are relatively high, this cell should be classified **at-risk**, warranting further risk mitigation. For example, the network might broadcast advisory messages to the affected UEs: *“Area A is at risk of COVID-19: It has many actively moving people.”*

**Fig. 2.**
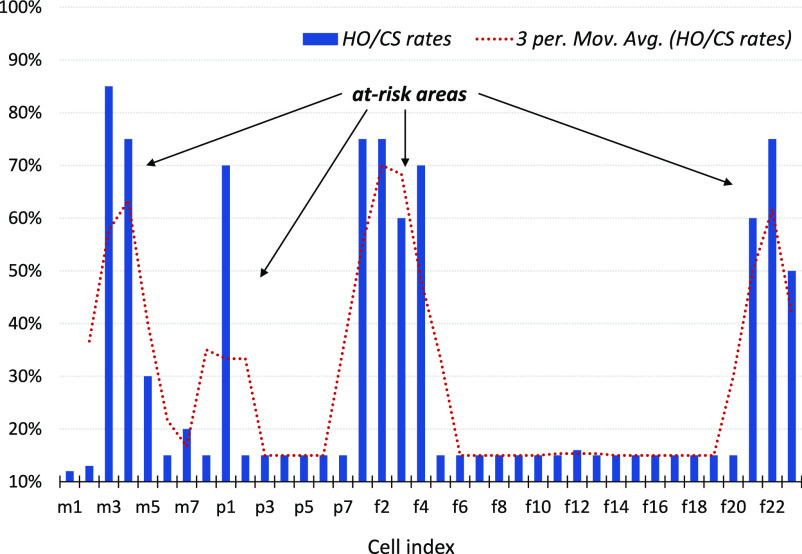
Illustration of *HO/CS* rates in regions with varying density and mobility.

For illustration, Fig. 2 shows that the *HO/CS* rates are higher in busy areas than in areas with low UE density/mobility. In this example, the following cells are **at-risk**: m3, m4, p1, f1, f2, f3, f4, f21, f22, and f23 (also labeled in Fig. 1). When people tend to stay home for a period of time, the corresponding *HO/CS* rates are lower than in crowded areas with high UE mobility (e.g., f8 and f10 in Fig. 2).

## IV. Discussion

A natural rule for deciding whether to classify an area as **at-risk** is to compare the measured *HO/CS* rate with a threshold value, prespecified according to the desired false-alarm probability. False alarms are not particularly problematic here because of the need to be conservative. Dire consequences can result from the presence of even a single carrier in an area with many actively moving people. While our strategy aims to identify areas with potentially high viral transmission, the *HO/CS* rates can also be used to estimate, for example, the percentage of people staying at home.

## V. Conclusion

We have introduced a new strategy for identifying areas that potentially contribute to the spread of COVID-19. Our strategy exploits existing cellular network procedures, *HO* and *CS*, required to maintain connectivity for mobile UEs. The frequency of *HO/CS* events reflects how the UEs move and gather within the coverage area. High *HO/CS* rates imply **at-risk** areas—those with high UE density and mobility over time. Measuring *HO/CS* rates allows distinguishing high- from low-risk areas, enabling prioritization of further risk mitigation.

## Supplementary Materials

Supplementary materials
